# Differential mosaicism of recombinant foot-and-mouth disease viruses resulting from heterologous superinfection of cattle

**DOI:** 10.1128/jvi.02213-24

**Published:** 2025-02-11

**Authors:** Carolina Stenfeldt, Ian H. Fish, Monica Rodriguez-Calzada, Gisselle Medina, Juergen A. Richt, Jonathan Arzt

**Affiliations:** 1 Foreign Animal Disease Research Unit, Agricultural Research Service, U.S. Department of Agriculture, Plum Island Animal Disease Center, Greenport, New York, USA; 2 Department of Diagnostic Medicine/Pathobiology, College of Veterinary Medicine, Kansas State University 70725 , Manhattan, Kansas, USA; 3 PIADC Research Participation Program, Oak Ridge Institute for Science and Education 17215 , Oak Ridge, Tennessee, USA; 4 Foreign Animal Disease Research Unit, Agricultural Research Service, U.S. Department of Agriculture, National Bio-and Agro Defense Facility, Manhattan, Kansas, USA; University of Michigan Medical School, Ann Arbor, Michigan, USA

**Keywords:** foot-and-mouth disease virus, FMDV, FMD, virus, virology, recombination, cattle, pathogenesis

## Abstract

Evidence from both field and experimental studies suggests that recombination is a common feature in the evolution of foot-and-mouth disease virus (FMDV). Recent studies have demonstrated that heterologous superinfection of cattle persistently infected with FMDV leads to rapid generation of inter-serotypic recombinant viruses in the upper respiratory tract mucosa. The current study demonstrates that the order of exposure to FMDV strains A24 Cruzeiro and O1 Manisa substantially influenced the patterns of mosaicism of resultant recombinants. FMDV recombinants were isolated from oropharyngeal fluid samples from 7 of 12 cattle following heterologous superinfection at 21 days post-initial infection. There was no apparent competitive advantage of either parental virus. However, recombinant viruses recovered from six of seven animals had gained, or regained through multiple recombination events, the capsid-coding sequence of FMDV O1 Manisa despite the presence of high titers of neutralizing antibodies against that virus. Additionally, a sub-genomic region of high amino acid diversity, spanning the 3′ portion of 3A through 3B, was derived from FMDV A24 in most of the recovered recombinants. Despite the frequent recovery of FMDV recombinants from the upper respiratory tract of superinfected animals, there was no detection of recombinant viruses in blood or lesions in the subset of animals that developed clinical foot-and-mouth disease during superinfection. Overall, these findings confirm the high frequency at which FMDV recombination occurs when persistently infected carrier cattle are exposed to a heterologous virus and reaffirm that superinfection of carriers should be considered as a source of FMDV genetic diversity in endemic regions.

**IMPORTANCE:**

Foot-and-mouth disease virus (FMDV) is a pathogen of domestic livestock with profound global socioeconomic impacts. FMDV causes a subclinical persistent infection in ruminant hosts, such as cattle, during which the animals may become sequentially infected by heterologous variants of the virus. Our previous works have demonstrated that such superinfections frequently lead to emergence of novel recombinant virus variants in the upper respiratory tracts of infected animals. This current investigation demonstrates that the order in which the animals are exposed to two different viruses substantially influences the structure of resultant recombinant genomes and confirms the frequency at which FMDV recombination occurs following heterologous superinfection of persistently infected FMDV carriers.

## INTRODUCTION

Foot-and-mouth disease (FMD) is a socioeconomically important, transboundary disease of cloven-hoofed livestock. The etiological agent is foot-and-mouth disease virus (FMDV), an *Aphthovirus* belonging to the *Picornaviridae* family (1).

Recent technological advances that have enabled cost-effective full-length sequencing of FMDV have demonstrated that recombination is a common feature of FMDV molecular evolution under natural conditions (2–4). Under laboratory conditions, FMDV recombination occurs at a high frequency in the upper respiratory tract of cattle that are exposed to two heterologous FMDVs, separated by 3–5 weeks, but not in animals that are simultaneously infected with the same two viruses (5–7). This finding suggests a potentially important role of persistently infected carrier animals in FMDV evolution and molecular epidemiology.

In cattle, the initial site of FMDV infection involves specific segments of epithelium in the nasopharyngeal mucosa (8–10). After establishment of primary infection, there is a phase of systemic generalization and viremia in clinically susceptible animals, coinciding with development of characteristic vesicular lesions in and around the oral cavity, and in areas of non-haired skin such as teats and coronary bands (11). In ruminants, FMDV is capable of causing a prolonged subclinical persistent infection in convalescent-phase animals that is associated with low-level viral replication restricted to the upper respiratory or gastrointestinal tract, depending on host species (reviewed in reference 12). In cattle, persistent FMDV infection has been localized to the same segments of nasopharyngeal epithelium that are the sites of primary infection (8–10). Animals that are protected against clinical FMD by prior exposure or vaccination may become subclinically infected (neoteric infection) upon exposure to the virus and progress to persistent infection without any detectable signs of disease (10, 12).

The FMDV carrier state has historically been defined by the ability to isolate FMDV from samples of oropharyngeal fluid (OPF), obtained by scraping of the oropharyngeal and nasopharyngeal mucosa using a probang cup (13, 14), beyond 28 days postinfection (dpi). More recent investigations have demonstrated that the divergence between cattle that clear infection and those that maintain persistent FMDV infection occurs earlier than previously assumed: around 14–21 dpi in naïve cattle and 7–14 dpi in vaccinated cattle (12). Although persistent FMDV infection can readily be detected in a large proportion of FMD-convalescent cattle through probang sampling, persistently infected carriers generally do not shed virus in oronasal secretions (12, 15, 16). The risk of contagion associated with FMDV carriers is therefore believed to be very low. Although a number of small-scale, experimental trials have failed to demonstrate transmission from persistently infected carrier cattle by direct contact exposure (16–18), it has been shown that unadulterated OPF obtained from FMDV carriers causes fulminant FMD if deposited into the upper respiratory tract of naïve cattle (19). Additionally, the presence of FMDV carriers is not tolerated in any country seeking official status as free of FMD (20).

Our previous work demonstrated that superinfection of cattle persistently infected with FMDV A24 Cruzeiro, with the heterologous FMDV strain O1 Manisa, led to rapid generation of inter-serotypic recombinant viruses in the upper respiratory tract of infected cattle as early as 48 h after infection (5–7). All recovered recombinant viruses had capsid-coding regions derived from the most recent virus exposure (FMDV O1 Manisa), suggesting a selective advantage associated with an initial lack of immunity against the capsid of the superinfecting virus. By contrast, no recombinant viruses were recovered from cattle that had been simultaneously infected with the same two virus strains (5).

This current work is a continuation of our previous work studying FMDV coinfections in cattle under controlled experimental conditions. The output of this work confirms a high frequency of viral recombination occurring in the upper respiratory tract of superinfected FMDV carrier cattle while demonstrating that the specific order of virus exposures significantly influences the patterns of mosaicism of resultant recombinants.

## RESULTS

### Phase 1

**Fig 1:**
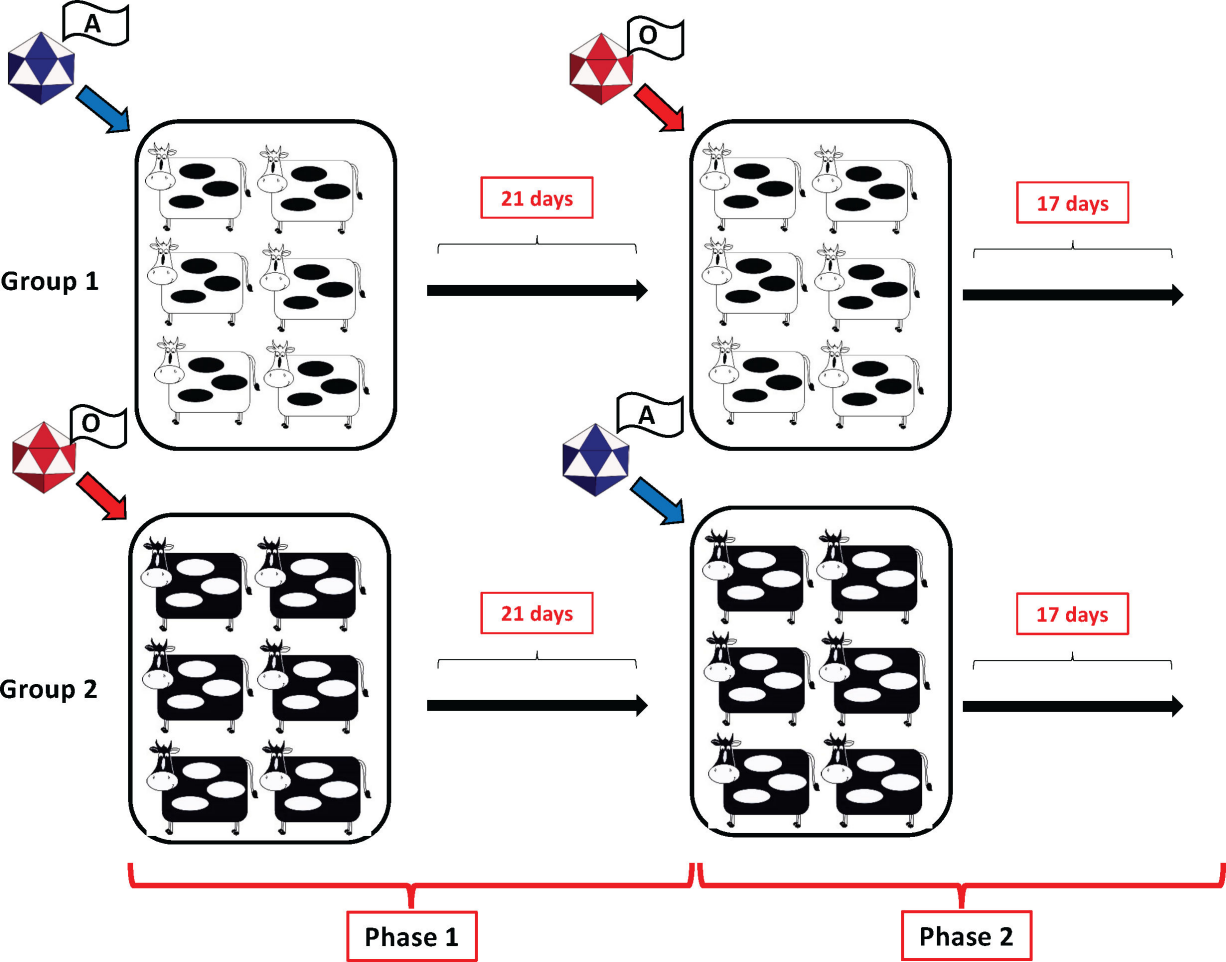
Experimental design. Two groups of six cattle each were infected with either FMDV A24 Cruzeiro (group 1) or FMDV O1 Manisa (group 2), via intra-nasopharyngeal deposition of inoculum on study day 0. On study day 21, both groups of cattle were superinfected with the heterologous virus. Phase 1 refers to study days 0–20, and phase 2 comprises days 21–38.

In the first phase of the experiment, two groups of six cattle each were infected with 10^6^TCID_50_ of FMDV via intra-nasopharyngeal deposition (Fig. 1). Group 1 animals were infected with FMDV A24 Cruzeiro (FMDV A24), while animals in group 2 were infected with FMDV O1 Manisa (FMDV O1M; Fig. 1).

**Fig 2:**
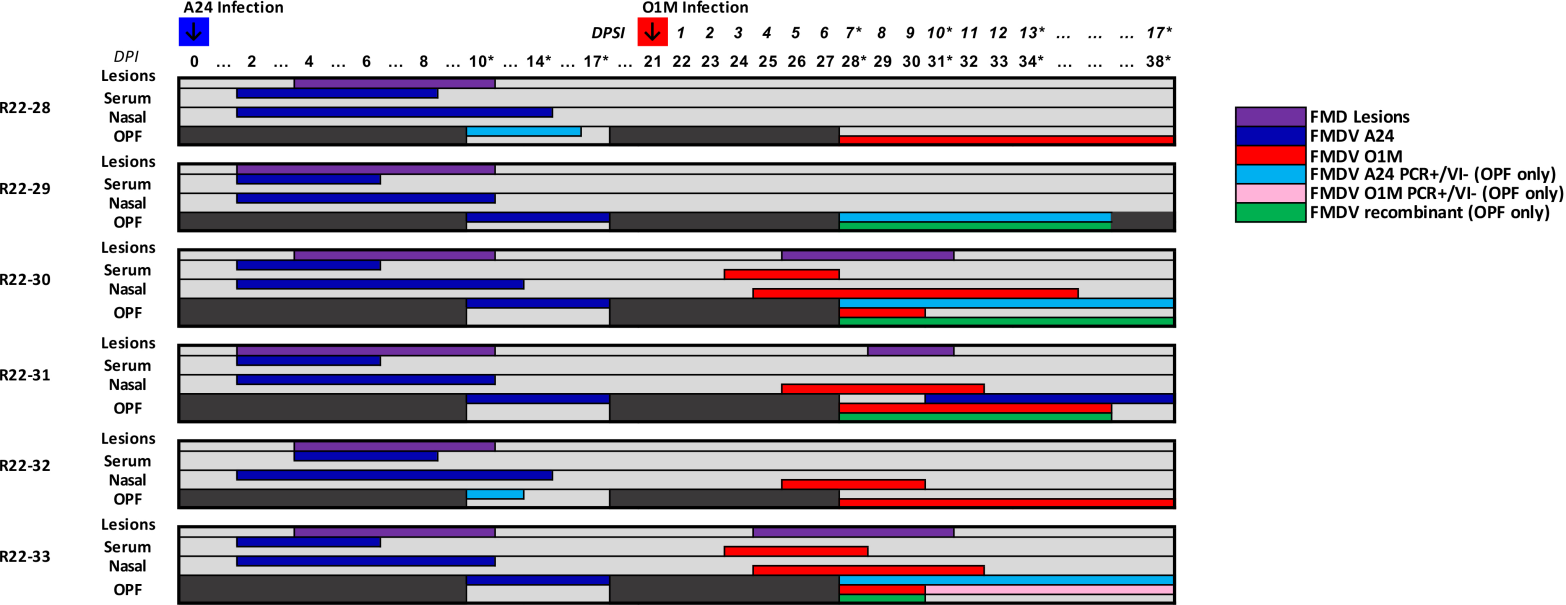
Strain-specific detection of FMDV A24 and O1M in antemortem samples, group 1. Group 1 cattle were infected with FMDV A24 on day 0 (blue arrow) and FMDV O1M on day 21 (red arrow). Colored bars in the chart represent the duration (days) of detection of FMDV RNA from A24 (blue) and O1M (red) by strain-specific qRT-PCR in serum and nasal swabs for each of the six cattle. Purple bars represent the presence of FMDV vesicles (based on clinical examinations performed from 0 to 10 days post-initial infection and superinfection). FMDV detection in oropharyngeal fluid OPF) samples was performed by strain-specific qRT-PCR and virus isolation (VI). For OPF samples, blue (FMDV A24) and red (FMDV O1M) bars indicate that samples were positive by both qRT-PCR and VI, with strain-specific RNA detection in un-passaged OPF as well as VI supernatants. Light blue (FMDV A24) and pink (FMDV O1M) bars indicate that OPF samples were positive by strain-specific qRT-PCR but negative for the corresponding virus strain by VI. Green bars indicate the presence of recombinant FMDVs as determined by NGS. DPI, days postinfection. DPSI, days post superinfection. Numbered days correspond to days on which nasal swabs and blood samples were collected. Asterisks indicate sample days on which OPF samples were collected by probang sampling.

**Fig 3:**
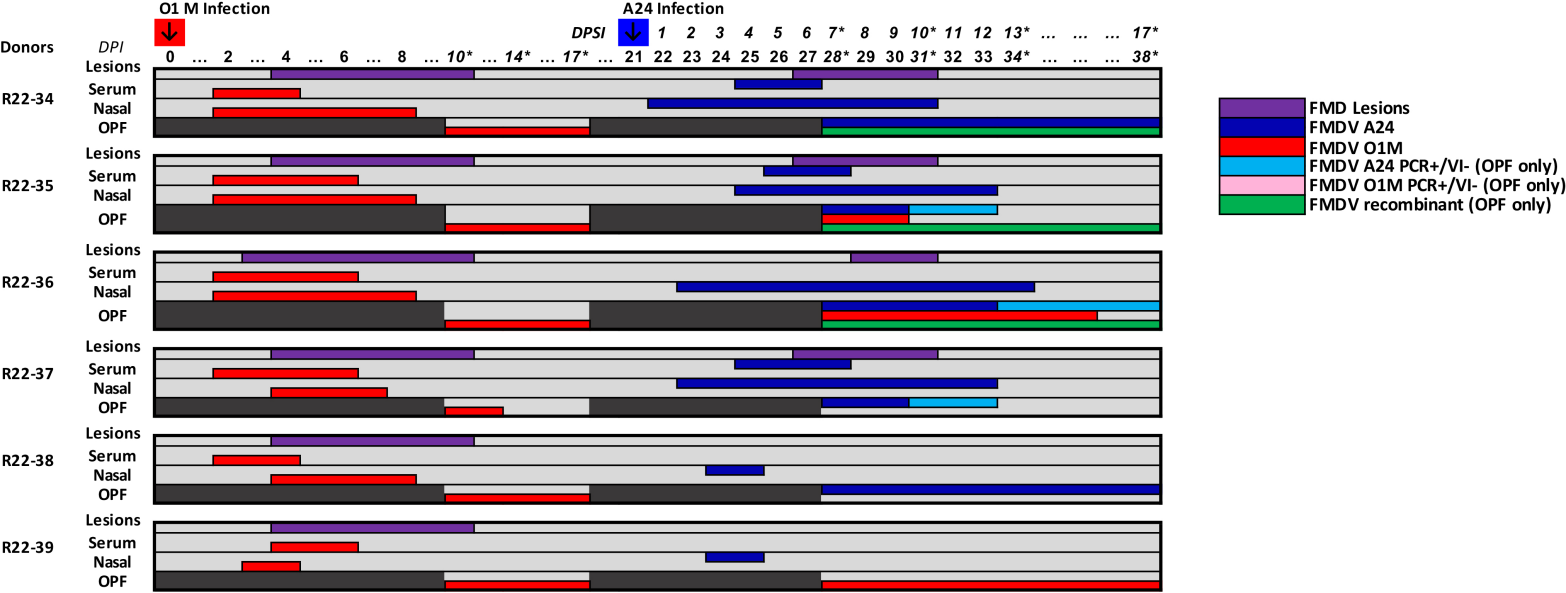
Strain-specific detection of FMDV A24 and O1M in antemortem samples, group 2. Group 2 cattle were infected with FMDV O1M on day 0 (red arrow) and FMDV A24 on day 21 (blue arrow). Colored bars in the chart represent the duration (days) of detection of FMDV RNA from A24 (blue) and O1M (red) by strain-specific qRT-PCR in serum and nasal swabs for each of the six cattle. Purple bars represent the presence of FMDV vesicles (based on clinical examinations performed from 0 to 10 days post-initial infection and superinfection). FMDV detection in oropharyngeal fluid (OPF) samples was performed by strain-specific qRT-PCR and virus isolation (VI). For OPF samples, blue (FMDV A24) and red (FMDV O1M) bars indicate that samples were positive by both qRT-PCR and VI, with strain-specific RNA detection in un-passaged OPF as well as VI supernatants. Light blue (FMDV A24) and pink (FMDV O1M) bars indicate that OPF samples were positive by strain-specific qRT-PCR but negative for the corresponding virus strain by VI. Green bars indicate the presence of recombinant FMDVs as determined by NGS. DPI, days postinfection. DPSI, days post superinfection. Numbered days correspond to days on which nasal swabs and blood samples were collected. Asterisks indicate sample days on which OPF samples were collected by probang sampling.

All cattle in both study groups developed clinical FMD of expected severity and duration, with vesicular lesions detected in the oral cavities and/or on feet starting between 2 and 4 days postinfection. Similarly, all animals developed viremia, defined by detection of FMDV RNA in serum samples, of approximately 3–7 days duration, and shedding of FMDV RNA was consistently detected in nasal swabs (Fig. 2 and 3). In study group 1, four of six cattle maintained infection with FMDV A24 throughout phase 1 of the experiment, determined by isolation of virus from oropharyngeal fluid samples on days 10 through 17. In study group 2, five of six animals maintained infection with FMDV O1M through phase 1 (Fig. 2 and 3).

### Phase 2

On study day 21, the 12 animals from phase 1 were superinfected with the heterologous virus via intra-nasopharyngeal deposition. Thus, cattle in group 1 were superinfected with FMDV O1 Manisa, and the animals in group 2 were superinfected with FMDV A24 (Fig. 1).

The clinical outcome in superinfected cattle varied across individuals and cohorts. In group 1, three of six cattle developed clinical FMD after the second virus exposure (Fig. 2). Viremia was only detected in two of the three animals with FMD lesions, whereas the third clinical animal (ID R22-31) had only a single vesicular lesion on the dental pad without concurrent viremia (Fig. 2). In group 2, which were initially infected with FMDV O1M and superinfected with FMDV A24, four of the six cattle developed clinical FMD, with concurrent viremia in three of the four clinically affected cattle and lesions limited to the dental pad in the non-viremic fourth animal (ID R22-36; Fig. 3).

### FMDV shedding and persistence in superinfected cattle

Cattle that developed clinical FMD following superinfection shed FMDV RNA in nasal secretions for longer durations and at greater quantities compared to superinfected cattle that did not develop clinical FMD (data not shown). The identity of the virus detected in nasal secretions always matched the superinfecting (most recent) virus (Fig. 2 and 3). Similarly, only the superinfecting (second) virus was recovered from phase 2 vesicular lesions. FMDV was detected in OPF samples from all superinfected animals when probang sampling was resumed at 7 days post-superinfection (dpsi; 28 dpi). All virus isolation-positive OPF samples were Illumina-sequenced in duplicate to confirm virus compositions. As expected, only the superinfecting virus was detected in phase 2 OPF samples from the three animals that had cleared infection during phase 1 (IDs R22-28, R22-32, R22-37). This was also the case for animal R22-38, whereas OPF samples from animal R22-39 contained only the virus from the initial infection. OPF samples obtained from the remaining seven superinfected cattle contained inter-serotypic recombinant viruses with or without concurrent detection of one or both parental viruses (Fig. 2 and 3; Table 1). The composition of samples that included multiple serotypes and inter-serotypic recombinant genomes was initially identified by Illumina sequencing and deep-sequence computational analysis. Additionally, plaque purification with subsequent Illumina sequencing was used to confirm constituent genomes in samples with complex virus populations (Table 2).

**TABLE 1:** Viruses present in phase 2 oropharyngeal fluid samples^*a*^

Study group	Animal ID	Order of infection		Day post superinfection (dpi)			
				7 (28)	10 (31)	13 (34)	17 (38)
1	R22-28	A ⇒	⇒ O	O^†^	O	O	O
	R22-29			Rec	Rec*	Rec	NA
	R22-30			O / Rec	Rec	Rec*	Rec
	R22-31			O / Rec	O / Rec*	O / A / Rec	A
	R22-32			O^†^	O	O	O
	R22-33			O / Rec	–	–	–
2	R22-34	O ⇒	⇒ A	A / Rec*	A / Rec	A / Rec	A / Rec
	R22-35			O / A / Rec	Rec*	Rec	Rec
	R22-36			O / A / Rec	O / A / Rec*	O / Rec	[O] / Rec
	R22-37			A^†^	–	–	–
	R22-38			A	A	A	A
	R22-39			O	O	O	O

^*a*^ O, FMDV O1M; A, FMDV A24; Rec, recombinant FMDV; ‘-‘, virus isolation negative sample (not sequenced); NA, not applicable (sample not collected); ^†^, animal had cleared the first virus prior to superinfection; *, recombinants confirmed by plaque isolation; Underscore, most abundant virus in samples with multiple viruses detected; ‘[ ]’, ambiguous result.

**TABLE 2:** Recombinant FMDVs isolated from plaques

Animal ID	DPI/DPSI	Plaques sequenced	Wild type	Recombinant haplogroups	Recombinant breakpoint loci
R22-29	31/10	16	No	2	2B: 4348-4361; 2C/3A: 5378-5421
R22-30	34/13	2	No	1	2C: 5209-5225
R22-31	31/10	10	FMDV O1M	1	3D: 7165-7175
R22-34	28/7	9	FMDV A24	1	3D: 6793-6794
R22-35	31/10	15	No	1	5′UTR: 687-705, 3A: 5650-5659, 3C: 6073-6075
R22-36	31/10	23	FMDV O1M	1	2C: 4927-4946, 3B: 5949-5953

### Characteristics of FMDV recombinants detected in OPF samples from superinfected cattle

#### Group 1

**Fig 4:**
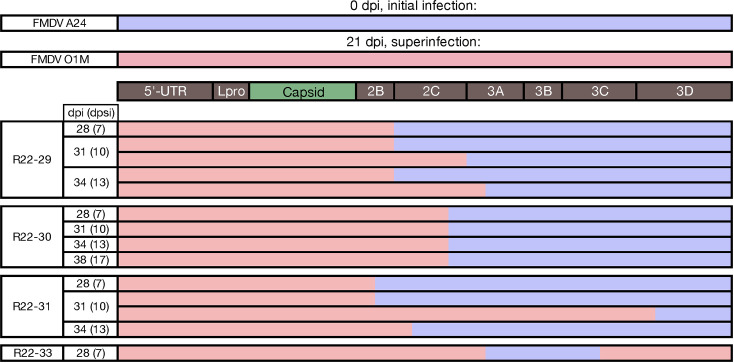
Mosaic genomes, group 1. Following FMDV O1M superinfection of cattle subclinically infected with FMDV A24, inter-serotypic recombinant genomes were identified through Illumina sequencing of oropharyngeal fluid samples. The composition of the mosaic viral genomes identified at each time point is illustrated, blue representing FMDV A24 parental derived segments and red representing FMDV O1M derived. Some time points include multiple viruses with different breakpoints (e.g., R22-29 31 dpi). dpi, days post (initial) infection; dpsi, days post superinfection.

**Fig 5:**
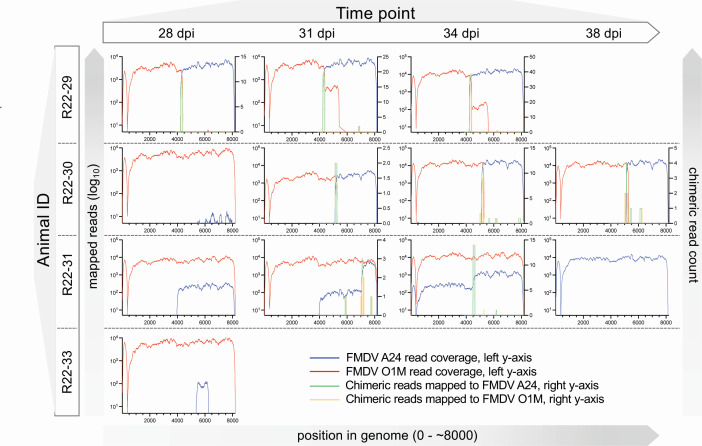
FMDV read coverage in samples with recombinant genomes detected, group 1. Mapped read coverage of each FMDV serotype isolated from oropharyngeal fluid sampled at 28–38 dpi from group 1 cattle (FMDV A24 infection on day 0, FMDV O1M infection on day 21) with evidence of inter-serotypic recombinant viruses. Blue and red lines indicate reads with highest identity to FMDV A24 and O1M, respectively. Left *y*-axis minimum is five reads. Reads mapping equally well to both reference genomes (chimeric reads [crf]s) remapped to each reference are also plotted; green and yellow lines reflect this coverage to the A24 and O1M references, respectively. The artefactual drop in coverage at the 5′ end of each genome (leftward end of the *x*-axis) is the site of the poly-C tract, for which Illumina sequencing and mapping typically fail. Note: Some graphs do not have crf data.

Inter-serotypic recombinants were detected in phase 2 OPF samples obtained from four cattle in group 1 (Fig. 2; Table 1). All recombinant viruses recovered from group 1 animals had capsid-coding regions derived from FMDV O1M, representing the second (superinfecting) virus (Fig. 4). In two animals (R22-29 and R22-30), recombinant viruses outcompeted the parental viruses and were the only viruses detected through the final sampling time points (Table 1). In samples from animal R22-29, three different inter-serotypic recombinants were present, with breakpoints in 2B and 2C/3A, respectively (Fig. 4; Table 2). The 2B recombinant was present across all time points through 34 dpi/13 dpsi, whereas the 2C/3A recombinants varied over time. The coverage was 10–100× greater for the recombinant with a breakpoint in the 2B coding region compared to the other recombinant viruses (Fig. 5). Samples from animal R22-30 contained a single recombinant virus with a breakpoint in the 2C coding region (Fig. 4 and 5). This recombinant was detected concurrent with the parental FMDV O1M virus on the first phase 2 OPF sampling time point and as the single dominant virus on subsequent sampling time points (Table 1). Animal R22-31 maintained persistent infection with the parental FMDV A24 throughout the study period. However, FMDV A24 was not detected by quantitative real-time RT-PCR (qRT-PCR) or virus isolation (VI) in the 28 dpi/7 dpsi OPF sample and was also not detected by NGS in the subsequent (31 dpi/10 dpsi) sample (Fig. 2; Table 1). Additionally, the superinfecting parental FMDV O1M was detected as the dominant constituent, concurrent with multiple recombinants with breakpoints in 2B, 2C, and 3D coding regions in samples obtained at 7, 10, and 13 dpsi (Fig. 4), whereas FMDV A24 outcompeted all recombinants to exist as the only virus detected at the final sampling time point at 17 dpsi/38 dpi (Fig. 5; Table 1). The OPF sample obtained at 7 dpsi/28 dpi from animal R22-33 contained the superinfecting FMDV O1M as the dominant virus together with low levels of a recombinant virus with breakpoints in both the 3A and 3C coding regions (Fig. 4 and 5). Subsequent OPF samples from this animal were positive for both FMDV A and O by strain-specific qRT-PCR assays but negative by virus isolation suggesting that the animal had cleared infection (Fig. 2).

#### Group 2

**Fig 6:**
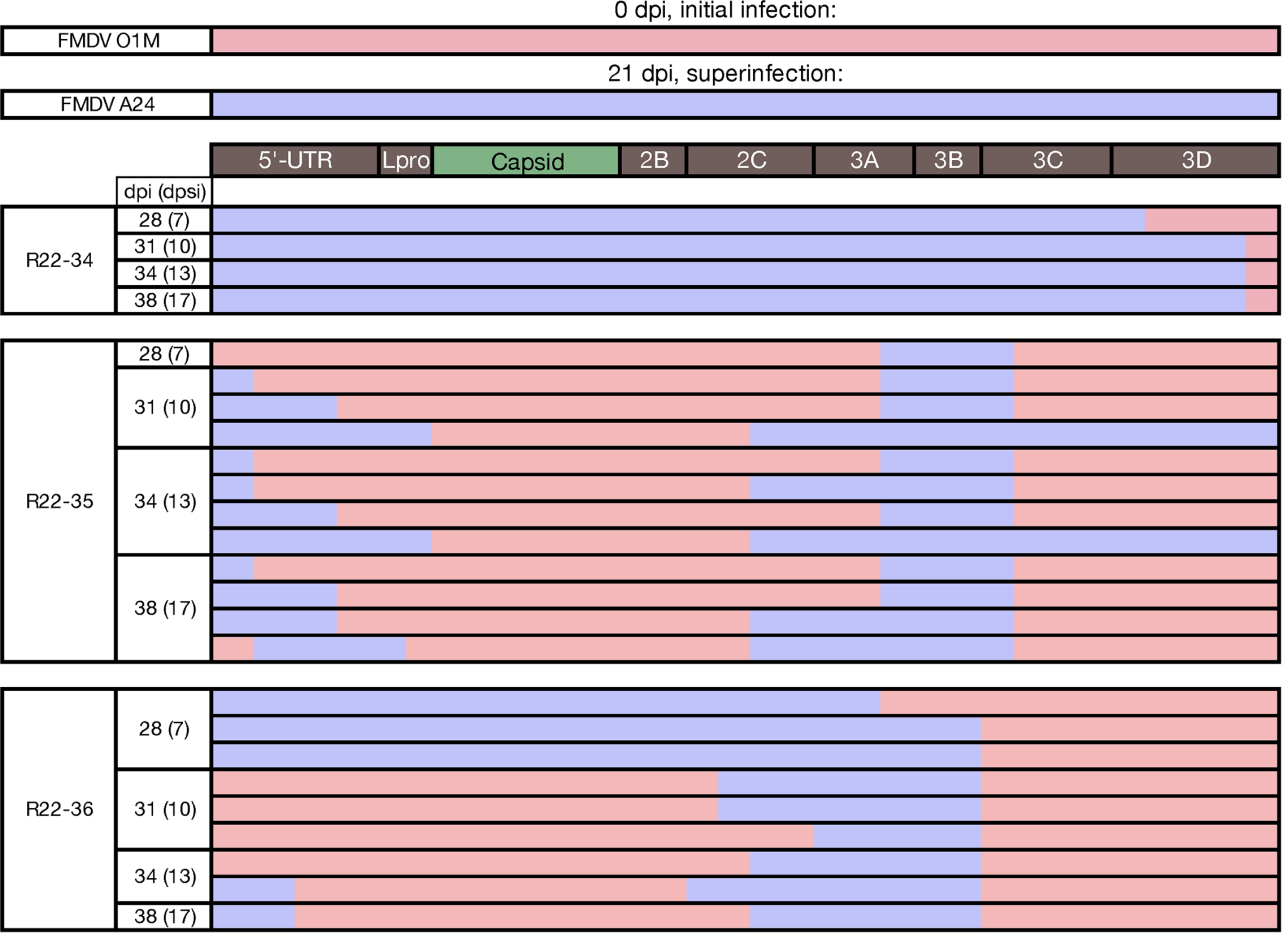
Mosaic genomes, group 2. Following FMDV A24 superinfection of cattle subclinically infected with FMDV O1M, inter-serotypic recombinant genomes were identified through Illumina sequencing of oropharyngeal fluid samples. The composition of these mosaic viral genomes identified at each time point is illustrated, red representing FMDV O1M parental derived segments and blue representing FMDV A24 derived. Many time points include multiple viruses with different breakpoints (e.g., R22-35 31 dpi). dpi, days post (initial) infection; dpsi, days post superinfection.

**Fig 7:**
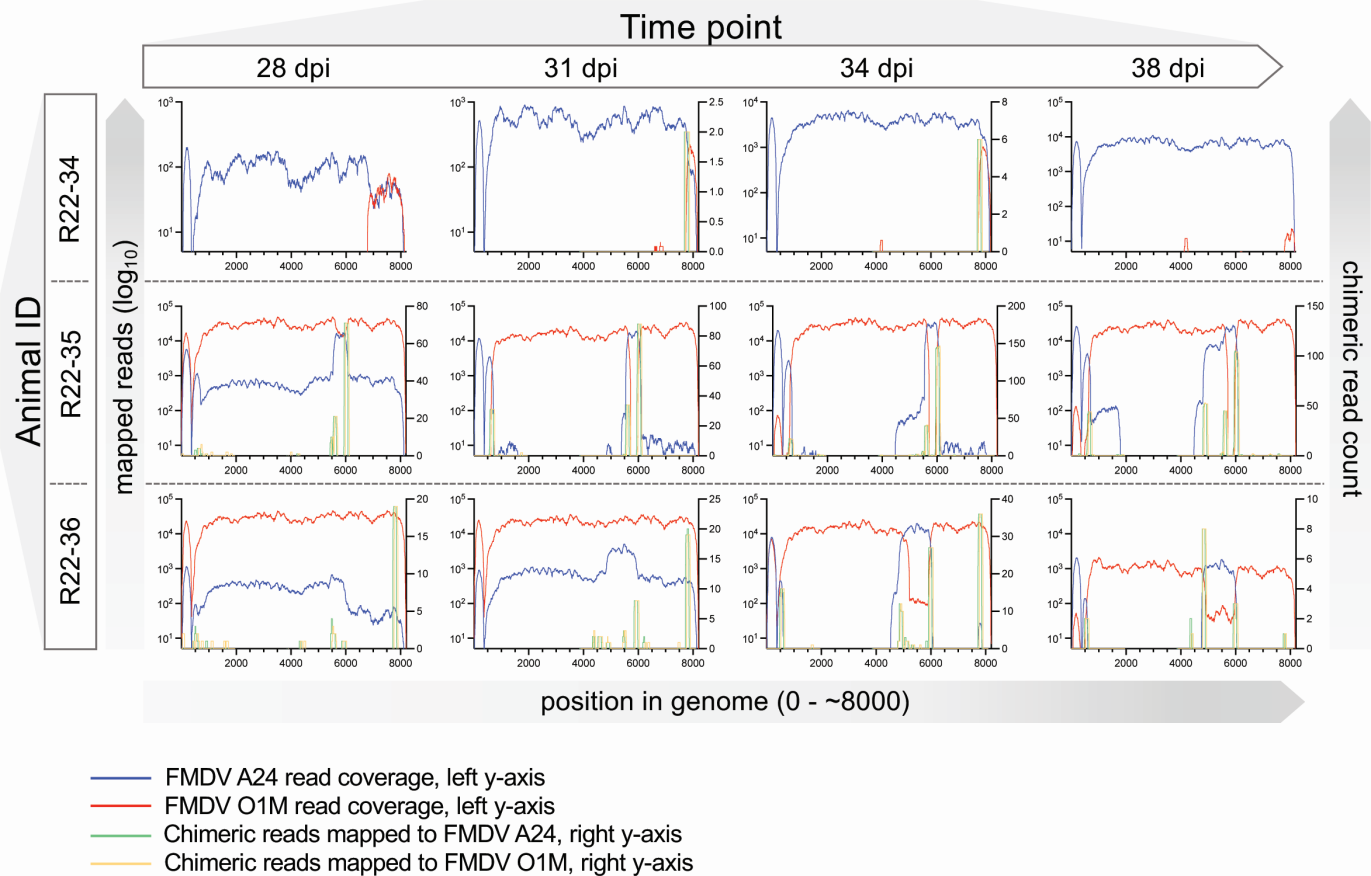
FMDV read coverage in samples with recombinant genomes detected, group 2. Mapped read coverage of each FMDV serotype isolated in oropharyngeal fluid sampled at 28–38 dpi from group 1 cattle (FMDV A24 infection on day 0, FMDV O1M infection on day 21) with evidence of inter-serotypic recombinant viruses. Blue and red lines indicate reads with highest identity to FMDV A24 and O1M, respectively. Left *y*-axis minimum is five reads. Reads mapping equally well to both reference genomes (chimeric reads [crf]s) remapped to each reference are also plotted; green and yellow lines reflect this coverage to the A24 and O1M references, respectively. The artefactual drop in coverage at the 5′ end of each genome (leftward end of the *x*-axis) is the site of the poly-C tract, for which Illumina sequencing and mapping typically fail. Note: Some graphs do not have crf data.

Recombinant viruses were recovered from phase 2 OPF samples of three animals in group 2, which were initially infected with FMDV O1M, and superinfected with FMDV A24. Samples from animal R22-34 contained the superinfecting FMDV A24 as the dominant virus as well as two different recombinant viruses for which most of the genome was derived from FMDV A24, with only the 3′ end of the 3D coding region derived from FMDV O1M (Fig. 6 and 7; Tables 1 and 2). All phase 2 OPF samples from animals R22-35 and R22-36 contained multiple recombinant viruses. Samples from earlier time points also contained one or both parental viruses, whereas only recombinant viruses were present at the final sampling time point for both of these animals. The recombinants detected in OPF samples from these two individuals were different from all other recombinant viruses in the study as they did not have the prevalent pattern of retaining the capsid-coding region derived from the superinfecting virus. Instead, these viruses were seemingly the result of multiple recombination events resulting in mosaic genomes with capsid-coding regions from the initial infection (FMDV O1M) and segments derived from the superinfecting virus (FMDV A24) within the 5′ UTR as well as in parts of nonstructural coding regions (Fig. 6 and 7; Tables 1 and 2). All major inter-serotypic recombinants recovered from phase 2 OPF samples from animals R22-35 and -36 included at least two breakpoints (e.g., in 5′-UTR, Lpro, and 3C). These patterns suggest that presumed earlier intermediate forms of these recombinants, which were not recovered from any collected samples, must have encoded capsids derived from the superinfecting FMDV A24, consistent with the recombination patterns observed in group 1 animals.

### Confirmation of detected recombinant viruses by plaque isolation

To confirm the inferred recombinant genomes and the computational methodology used for detection, plaque purification followed by Illumina sequencing was done on a subset of OPF samples. One OPF sample from each of the six animals in which recombinants represented >10% of the estimated virus population in the sample was analyzed (Table 2). In all cases, the major recombinant species isolated in plaques was consistent with the predicted breakpoints and genomic composition of the dominant recombinant that had been detected using computational tools. Similarly, the appropriate parental viruses were isolated from separate plaques in all instances when they were predicted to be present. Deep sequence analysis of the OPF samples prior to plaque isolation had suggested that many of the samples included multiple recombinants (i.e., genomes with different breakpoints). For example, sample R22-29 31 dpi OPF was predicted to include at least two different recombinants at an approximately 9:1 relative frequency. These viruses were both successfully isolated through plaque purification (Table 2), thereby confirming the computational approach.

### FMDV serology

**Fig 8:**
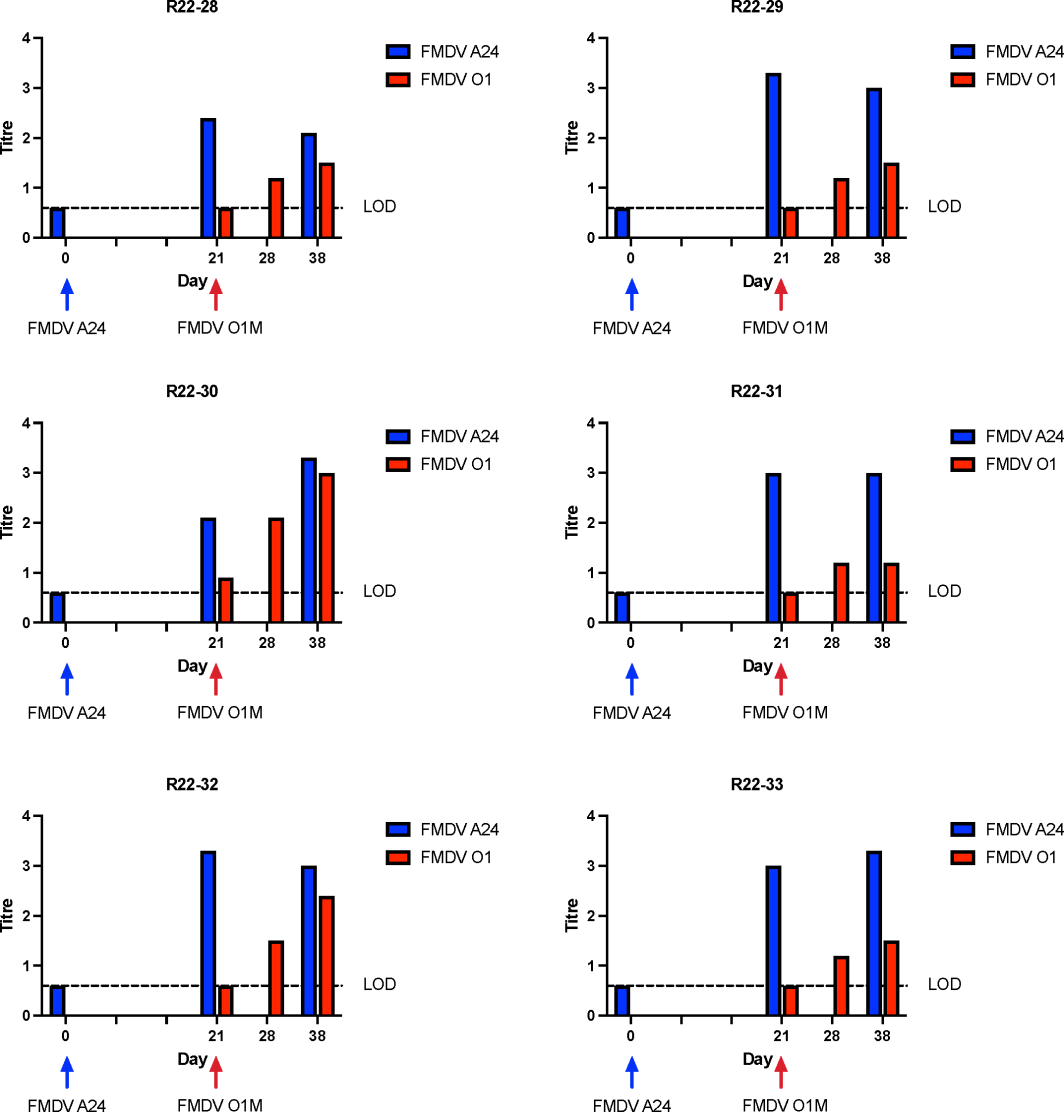
Neutralizing anti-FMDV titers in serum, group 1. Neutralizing titers in serum samples were measured against FMDV A24 on days 0, 21, and 38 and against FMDV O1M on days 21, 28, and 38. Titers were calculated according to the Spearman-Karber method and expressed as log10 of the reciprocal of the final serum dilution that neutralized 100 TCID_50_ of the respective serotype in 50% of the wells.

**Fig 9:**
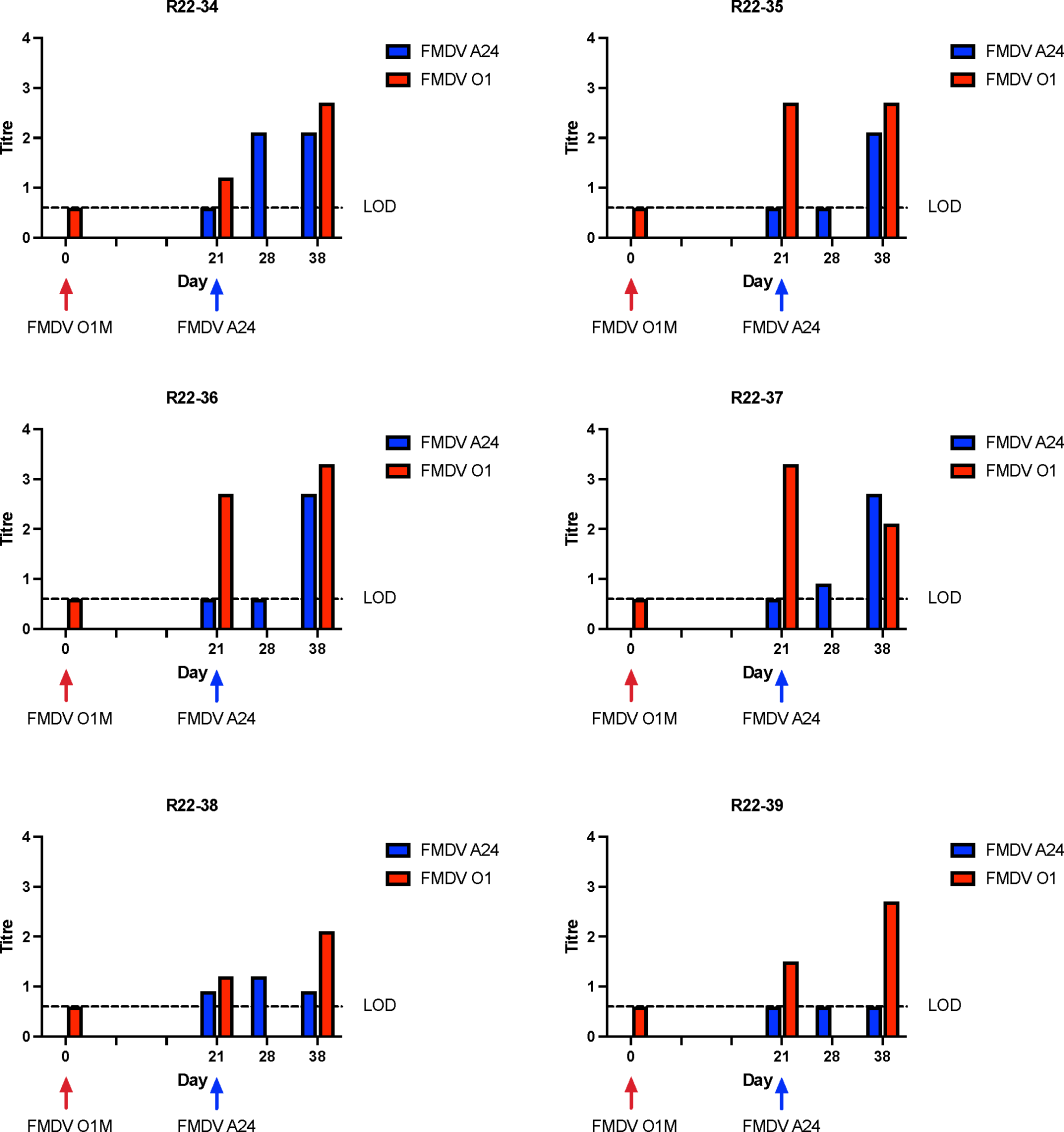
Neutralizing anti-FMDV titers in serum, group 2. Neutralizing titers in serum samples were measured against FMDV O1M on days 0, 21, and 38 and against FMDV A24 on days 21, 28, and 38. Titers were calculated according to the Spearman-Karber method and expressed as log10 of the reciprocal of the final serum dilution that neutralized 100 TCID_50_ of the respective serotype in 50% of the wells.

The serological responses to infection were assessed by serum neutralization against both challenge viruses. Serum obtained on 0, 21, and 38 dpi were tested against the initial infecting virus, and serum obtained on days 21, 28, and 38 (corresponding to 0, 7, and 17 dpsi) were tested against the superinfecting virus. Most animals had predictable responses with increasing titers against both viruses throughout the study period (Fig. 8 and 9). There were a few exceptions to that pattern: animal R22-39 did not seroconvert against FMDV A24 (Fig. 9), suggesting that this individual was not infected by the second virus exposure despite detection of low levels of FMDV RNA in nasal swabs following inoculation. Some individuals had declining antibody titers against the first virus on the final sampling day (38 dpi). Other noteworthy findings were a lack of detectable antibodies against the superinfecting virus (FMDV A24) at 28 dpi/7 dpsi in animals R22-35 and R33-36. These two individuals were the only animals with confirmed superinfection that did not have detectable neutralizing antibodies against the superinfecting virus at 7 dpsi. Interestingly, animals R22-35 and R33-36 were also the two individuals in which recovered recombinant viruses did not maintain capsid-coding regions derived from the superinfecting virus.

## DISCUSSION

Foot-and-mouth disease virus exists as multiple serotypes and strains that may co-circulate in endemic regions. The FMDV serotypes do not confer immunological cross-protection, and the existence of a prolonged subclinical carrier state in ruminant hosts facilitates overlapping infections by heterologous viruses (21). FMDV inter-serotypic recombinants are commonly discovered when full-length genome sequencing is used to analyze field samples (2, 3). However, this complexity of FMDV ecology is often overlooked as the majority of phylogenetic studies are based exclusively upon VP1 sequencing.

Subclinical FMDV infection of ruminants can be divided into temporally acute (neoteric) versus persistent infection. Although indistinguishable in field scenarios in which the time of virus exposure may not be known, a critical difference between these two stages of infection consists of notably different levels of virus shedding in oronasal secretions (22). Neoteric FMDV infection is more commonly associated with detectable virus shedding in the absence of clinical disease, whereas the FMDV carrier state is characterized by lack of shedding of virus in secretions, despite the presence of infectious virus in the upper respiratory tract (12, 15, 16).

The probability of FMDV transmission from (any) subclinically infected individual is likely magnitudes lower than the probability of transmission from a clinical FMD case, making such events exceedingly difficult to demonstrate under experimental conditions. There is a lack of experimental evidence of FMDV transmission from persistently infected cattle (16, 17, 23). Although historical events are often cited as evidence of the opposite, the sources of such information are anecdotal at best and seemingly outweighed by more comprehensive evidence describing lack of transmission from carrier cattle (24). However, more recent evidence from studies of herds of Asian buffalo (*Bubalus bubalis*) suggests that substantial circulation of FMDV does occur, within and between herds, in the absence of clinical FMD (21). Such subclinical circulation of virus is likely due to neoteric infections, with clinical protection conferred by repeated virus exposures and/or vaccination. Superinfected FMDV carriers are concurrently persistently and neoterically infected. Our previous studies demonstrated that recombinant FMDVs could be isolated from tissues of the upper respiratory tract as early as 48 h postinfection (7), which would facilitate potential shedding and transmission of recombinant viruses during subclinical neoteric superinfection of carriers.

The narrow cellular tropism that characterizes both the primary and persistent phases of FMDV infection (8, 10) promotes coinfection on a cellular level when persistently infected FMDV carriers are superinfected. Our previous *in vivo* studies demonstrated that FMDV recombination was exceedingly common when virus exposures were staggered by 3–5 weeks, but that recombination did not occur when animals were simultaneously infected with the same two viruses (5, 7). The precise mechanisms behind this are not known. However, it is known that while acute infection with FMDV induces an immediate cellular antiviral state (25), persistent FMDV infection is believed to suppress the antiviral response (26, 27). Thus, persistently infected epithelial cells may be more permissible to coinfection compared to cells that are acutely infected, which would explain why recombination does not occur during simultaneous superinfection (5, 7).

Our previous findings suggested that the apparent success of inter-serotypic recombinants during early superinfection was in part explained by the initial lack of neutralizing antibodies against the capsid of the superinfecting virus (5). However, in the current study, recombinant viruses from two individuals regained the capsid-coding region derived from the first virus within the first 7 days of superinfection. The presence of segments derived from the second virus within the 5′ UTR of those recombinants suggest that the viruses were the result of multiple recombination events, most likely involving an undetected intermediate recombinant virus with the full capsid-coding region derived from the second virus. It is noteworthy that while both individuals had high neutralizing antibody titers against the first virus at the time of superinfection, they were the only two animals that did not have detectable antibodies against the second virus at 7 days post superinfection. Thus, in this case, the serological response in these two animals seems to have been influenced by the occurrence of viral recombination favoring the capsid from the initial virus, rather than the other way around.

Recombination may occur stochastically in any coinfected cell. Yet, the mechanisms leading to an inter-serotypic recombinant virus becoming dominant and possibly outcompeting the parental viruses are not known. None of the recombinant viruses recovered from our current or previous studies had breakpoints within structural coding regions, suggesting that viruses derived from such recombination events would not be viable. The breakpoints within the nonstructural coding regions were quite varied and mapped to nearly all protein-coding segments from 2B to 3D. Most of the sub-genomic coding regions in which breakpoints were identified in this current study have previously been identified as sites of inter-lineage recombination in field-derived FMDVs of serotypes O, A, and Asia-1 (2, 28).

Analyses of recombinant genomes from the current study indicated that one segment derived from FMDV A24 was overrepresented in recombinants of both study groups; specifically, the 3′ portion of region 3A through 3B (Fig. 4 and 6). Most notably, this was also true for the recombinant viruses obtained from animals R22-35 and -36, which had regained the capsid-coding regions from FMDV O1M, in addition to also having most of the nonstructural coding regions derived from FMDV O1M. The pattern of apparent selection for this region of FMDV A24 was consistent with recombinant FMDVs recovered in previous studies using the same two parental virus strains (5, 6). Interestingly, this specific segment has the highest amino acid divergence between the two parental viruses across the entirety of the nonstructural protein-coding regions. It is also noteworthy that recombinant viruses obtained from six of the seven animals (across both groups) had capsid-coding regions derived from the FMDV O1M virus. The FMDV A and O capsids have slightly different physical characteristics, with the serotype O capsid known to be less stable at physiological pH. However, both strains used in the current investigation have similar virulence and clinical characteristics in cattle, which suggests that factors improving fitness at the cellular level may not correlate with phenotype at the animal level. Clearly, factors beyond the scope of the current investigation, such as the magnitude and characteristic features of the host’s innate immune response, may also influence cellular-level pathogenesis. The FMDV A24 virus that was used as inoculum in the current investigation encodes a noncanonical SGD motif in the receptor-binding region, which has been known to revert to the canonical RGD motif during the transition from acute to persistent infection (29). It is possible that there may have been some selective advantage of the canonical receptor-binding motif of the FMDV O1M capsid over the noncanonical SGD motif in animals that were superinfected with the FMDV A24 virus. It is, however, noteworthy that no such advantage was detected in previous studies in which cattle were simultaneously infected with both virus strains (5). Further experiments using different strains of FMDV could potentially clarify possible selective pressures driving FMDV recombination. Additionally, field surveys targeting sampling of cattle with subclinical FMDV infection, including indigenous breeds with reduced sensitivity to the clinical manifestations of FMD, may improve our understanding of the extent to which FMDV recombination occurs when persistently infected carrier cattle are exposed to heterologous FMDV strains under natural conditions.

### Conclusions

The current investigation confirms previous findings of abundant inter-serotypic recombination within the upper respiratory tract of FMDV carrier cattle upon superinfection with a heterologous virus. This experimental model represents a scenario in which FMDV carriers are simultaneously persistently and neoterically infected. There was greater variation in mosaicism of recovered recombinant genomes when the order of exposure to the different viruses was reversed, suggesting possible selection for specific segments of distinct viral genomes. The selective forces driving FMDV recombination are not known but likely include some selective pressure from the host response to infection as well as intrinsic advantages of specific viral genome segments. Specifically, the apparent lack of antiviral activation of infected epithelial cells within the bovine nasopharyngeal mucosa during persistent but not acute infection may be an important driver of FMDV recombination. Improved understanding of the natural occurrence of FMDV recombination and the implications on FMDV evolution will likely increase as full genome sequencing becomes more available and cost-efficient. The combined output of work from our laboratory in recent years suggests that superinfection of FMDV carrier cattle may represent a substantial source of global FMDV diversity. Elucidation of mechanisms of FMDV evolution in field and laboratory settings will contribute toward enhanced understating of molecular epidemiology and may ultimately improve FMD control and eradication.

## MATERIALS AND METHODS

### Viruses

The viruses used were bovine-derived isolates of FMDV O1 Manisa and FMDV A24 Cruzeiro. The pathogenesis of both virus isolates in cattle has been characterized in detail previously (5, 12, 30). The inoculation doses (10^6^ TCID_50_) were determined through titration of both virus stocks on LFBK-avb6 cells (31, 32).

### Animals and study design

This work is based on an animal experiment involving 12 cattle, carried out at the Plum Island Animal Disease Center, New York, USA. The cattle used were approximately 10-month-old Holstein heifers procured from a USDA-certified vendor.

The experiment included two groups of six cattle each. On day 0, study group 1 was infected with FMDV A24 through intra-nasopharyngeal deposition, while group 2 was infected with FMDV O1M using a similar route and dose. On study day 21, corresponding to the start of phase 2 of the experiment, all 12 cattle were superinfected with the heterologous virus. Thus, cattle in group 1 were superinfected with FMDV O1M, while the animals in group 2 were superinfected with FMDV A24 (Fig. 1). All inoculations were done using a total dose of 10^6^ TCID_50_ per animal. Animals were monitored and sampled through study day 38, which corresponded to 17 days after superinfection (dpsi; Fig. 1). The experiment involved another 12 contact-exposed cattle that are not described further herein.

### Sample collection

Samples collected for detection of FMDV consisted of whole blood obtained through jugular venipuncture, nasal swabs, and oropharyngeal fluid obtained through probang sampling (13, 14). Blood samples and nasal tampons were centrifuged for extraction of serum and nasal secretions. OPF samples were homogenized using a 16G, 6″ steel cannula attached to a syringe. Additionally, one aliquot of OPF designated for subsequent virus isolation was treated with 1,1,2-trichlorotrifluoroethane (TTE) for dissociation of immune complexes prior to freezing. All samples were aliquoted and stored at −70°C until further processing.

Through phase 1 of the experiment, blood and nasal swabs were collected every other day through days 0–10, and again at days 14 and 17, with OPF samples collected on days 10, 14, and 17. In the second phase of the study, sample collection consisted of daily nasal swabs on days 21–38, daily blood samples on days 21–30, and again on day 38, as well as OPF samples on days 28, 31, 34, and 38.

Clinical examinations were done under sedation to enable thorough inspection of feet and oral cavities. The animals were sedated for clinical exams every other day after each of the inoculations, until full lesion scores were recorded or through a maximum of 10 days. Lesion scores were calculated so that FMD lesions on the muzzle or in the oral cavity contributed one point, with one additional point counted for lesions on each foot, leading to a maximum score of 5. Samples of vesicle epithelium or vesicular fluid were obtained from sedated animals during the clinical examinations. The two study groups were handled by separate teams to avoid potential cross-contamination between the groups.

### FMDV RNA detection

Tissue samples (vesicle epithelium) were individually thawed and macerated using a TissueLyser bead beater (Qiagen, Valencia, CA). Fifty microliters of tissue macerate was transferred to 96-well plates, and samples were subjected to qRT-PCR analysis as follows.

All tissue macerates, serum, nasal fluid, and OPF samples were analyzed using qRT-PCR, targeting the 3D region of the FMDV genome (33) with forward and reverse primers adapted from Rasmussen et al. (34), and chemistry and cycling conditions as previously described (35). Additionally, two strain-specific qRT-PCR systems were used to differentiate between the two viruses used in the study. These systems were based on the same chemistry and cycling conditions as the pan-serotype 3D-targeting system but with primers and probes targeting the capsid-coding regions (5). All assays were run in parallel (i.e., extracted RNA was simultaneously analyzed using the three distinct detection systems). Cycle threshold values <38 were considered positive.

### Virus isolation

Aliquots of macerated epithelial samples and TTE-treated probang samples were cleared of debris and potential bacterial contamination by centrifugation through Spin-X filter columns (pore size 0.45 µm, Sigma-Aldrich). Virus isolation was performed using LFBK-αvβ6 cells (31, 36), following a protocol previously described (37). The presence of amplified FMDV in VI supernatants was confirmed by qRT-PCR (universal and strain-specific systems).

### Plaque isolation

Plaques were isolated from six passaged phase 2 OPF samples from animals R22-29 31d (16 plaques), R22-30 34d (3), R22-31 31d (16), R22-34 28d (10), R22-35 31d (15), and R22-36 31d (25). Plaque isolation was performed as previously described (6). In brief, supernatant from each OPF sample was serially diluted in media and inoculated on confluent LFBK-αvβ6 monolayer plates. At 24–28 h postinoculation, distinct plaques were picked into 500 mL PBS and stored at −70°C. Viral RNA extraction and Illumina library prep and sequencing were performed as described below.

### FMDV sequence acquisition

Illumina sequencing of full-length FMDV genomes was performed as described previously (5). Briefly, viral RNA was extracted from raw samples (vesicles and plaques) or supernatant from LFBK-αvβ6 cells after single passage (serum, nasal, or OPF samples) using the MagMAX RNA isolation kit (Thermo Fisher). DNA was depleted (DNA-free DNase kit; Ambion) followed by ds-cDNA synthesis using Superscript II (Invitrogen) and NEBNext Ultra II nondirectional RNA second-strand synthesis module (New England BioLabs). Illumina Nextera XT libraries were constructed, with each sample barcoded in duplicate. Sequencing was completed on the NextSeq 550 platform with the 300-cycle kit (2 × 150 bp, paired-end).

### Sequence analysis

Illumina reads were paired, trimmed, and filtered for quality and length using CLC Genomics Workbench v. 21-22. Reads were next mapped to each reference genome corresponding to the two inocula, FMDV A24 Cruzeiro (GenBank #AY593768) and FMDV O1 Manisa (#AY593823). The accuracy of basecalls and serotype-specific coverage were verified across replicate samples. For all phase 2 OPF samples, reference-specific coverage was plotted using GraphPad Prism 9.5 (www.graphpad.com).

Recombinant genomes were initially identified by detection of commensurate changes in read mapping to both reference (parental) genomes. These interchanges in genome coverage represented breakpoint loci where the genome(s) shifted from matching one parental sequence to the other. In order to infer the genomic composition of species present in these mixed samples, a combination of computational methods was used including haplotype reconstruction (CliqueSNV (38), analysis of relative genome coverage, detection of chimeric reads spanning presumed breakpoints, as well as analysis of genomes detected in serial samples from the same animal.

Computational investigation of inter-serotypic recombination included chimeric read analysis in CLC. Chimeric reads are those for which half of the sequence shares identity with one reference (parental inoculum) and the other half shares identity with the other. For each sample, chimeric reads were collected and then remapped to each reference independently. Chimeric reads mapping to both references at the same location were individually confirmed. This chimeric read analysis method can be considered a conservative approach to illustrate breakpoints in recombinant genomes because it only compiles reads (~135 bp post-trim) wherein the breakpoint is centralized.

### Haplotype reconstruction

The haplotype reconstruction program CliqueSNV (38) was used to assemble genome segments present at minority proportions within samples. While not used alone, this analysis provided additional evidence for lower frequency inter-serotypic recombinants in complex samples.

### FMDV serology

FMDV-neutralizing antibody titers against the initial virus (FMDV A24 for group 1 and FMDV O1M for group 2) were determined in serum samples obtained on study days 0, 21, and 38 and against the superinfecting virus (FMDV O1M for group 1 and FMDV A24 for group 2) on days 21, 28, and 38. Serum samples were heat-inactivated for 30 min at 56°C and used in a microtiter neutralization assay. Serial fourfold dilutions of serum (in MEM with 25 mM HEPES) on 96-well plates were incubated with 100 TCID_50_ of FMDV A24 or FMDV O1M for 1 h at 37°C and 5% CO_2_. Freshly trypsinized LFBK-αVβ6 cells were resuspended in MEM with 25 mM HEPES, 4 × 10^4^ cells/well were added to the plates, and the plates were incubated for another 72 h at 37°C and 5% CO_2_ before cytopathic effect was evaluated visually. Titers were calculated using the Spearman-Karber method and expressed as log10 of the reciprocal of the final serum dilution that fully neutralized the virus in 50% of replicate wells.

## Data Availability

The Sequence Read Archive (SRA) data have been submitted to NCBI under BioProject accession PRJNA1026883.
